# Development of a Non-invasive Device for Swallow Screening in Patients at Risk of Oropharyngeal Dysphagia: Results from a Prospective Exploratory Study

**DOI:** 10.1007/s00455-018-09974-5

**Published:** 2019-01-05

**Authors:** Catriona M. Steele, Rajat Mukherjee, Juha M. Kortelainen, Harri Pölönen, Michael Jedwab, Susan L. Brady, Kayla Brinkman Theimer, Susan Langmore, Luis F. Riquelme, Nancy B. Swigert, Philip M. Bath, Larry B. Goldstein, Richard L. Hughes, Dana Leifer, Kennedy R. Lees, Atte Meretoja, Natalia Muehlemann

**Affiliations:** 1 Swallowing Rehabilitation Research Laboratory, Toronto Rehabilitation Institute – University Health Network, 550 University Avenue, 12th floor, Toronto, M5G2A2 Canada; 2 Department of Speech-Language Pathology, Rehabilitation Sciences Institute, Faculty of Medicine, University of Toronto, Toronto, Canada; 3 Cytel, Cambridge, MA USA; 4 VTT, Espoo, Finland; 5 Medical Devices, Nestlé Health Science, Lausanne, Switzerland; 6 Marianjoy Rehabilitation Hospital, Wheaton, IL USA; 7 Regions Hospital, St. Paul, MN USA; 8 Boston Medical Center, Boston, MA USA; 9 New York-Presbyterian Brooklyn Methodist Hospital, Brooklyn, NY USA; 10 Department of Speech-Language Pathology, New York Medical College, Valhalla, NY USA; 11 Baptist Health Lexington, Lexington, KY USA; 12 Swigert & Associates, Inc., Biltmore Lake, NC USA; 13 Division of Clinical Neuroscience, Stroke Trials Unit, University of Nottingham, Nottingham, UK; 14 Department of Neurology, University of Kentucky, Lexington, KY USA; 15 Department of Neurology, University of Colorado Denver, Denver, CO USA; 16 Department of Neurology, Weill Cornell Medical College, New York, NY USA; 17 Institute of Cardiovascular and Medical Sciences, University of Glasgow, Glasgow, UK; 18 Department of Neurology, Helsinki University Hospital, Helsinki, Finland

**Keywords:** Deglutition, Deglutition disorders, Dysphagia, Swallowing, Screening, Devices

## Abstract

Oropharyngeal dysphagia is prevalent in several at-risk populations, including post-stroke patients, patients in intensive care and the elderly. Dysphagia contributes to longer hospital stays and poor outcomes, including pneumonia. Early identification of dysphagia is recommended as part of the evaluation of at-risk patients, but available bedside screening tools perform inconsistently. In this study, we developed algorithms to detect swallowing impairment using a novel accelerometer-based dysphagia detection system (DDS). A sample of 344 individuals was enrolled across seven sites in the United States. Dual-axis accelerometry signals were collected prospectively with simultaneous videofluoroscopy (VFSS) during swallows of liquid barium stimuli in thin, mildly, moderately and extremely thick consistencies. Signal processing classifiers were trained using linear discriminant analysis and 10,000 random training–test data splits. The primary objective was to develop an algorithm to detect impaired swallowing safety with thin liquids with an area under receiver operating characteristic curve (AUC) > 80% compared to the VFSS reference standard. Impaired swallowing safety was identified in 7.2% of the thin liquid boluses collected. At least one unsafe thin liquid bolus was found in 19.7% of participants, but participants did not exhibit impaired safety consistently. The DDS classifier algorithms identified participants with impaired thin liquid swallowing safety with a mean AUC of 81.5%, (sensitivity 90.4%, specificity 60.0%). Thicker consistencies were effective for reducing the frequency of penetration–aspiration. This DDS reached targeted performance goals in detecting impaired swallowing safety with thin liquids. Simultaneous measures by DDS and VFSS, as performed here, will be used for future validation studies.

**Electronic supplementary material:**

The online version of this article (10.1007/s00455-018-09974-5) contains supplementary material, which is available to authorized users.

## Introduction

Individuals with dysphagia are faced with two functional concerns: 1, the inability to swallow *safely*, whereby material enters the airway (“penetration–aspiration” [1]); and/or 2, the inability to swallow *efficiently*, with residue remaining in the pharynx [2, 3]. Impaired swallowing safety is associated with pneumonia [4] whereas impaired efficiency contributes to the risk of malnutrition [2, 5, 6] and of aspirating residue after the swallow [7]. Dysphagia is estimated to affect 6.7% of hospitalized patients in the United States, with an annual attributable cost of $547 million [8]. The burden of dysphagia is significant, as shown in a recent analysis of the United States Agency for Healthcare Research and Quality (AHRQ) Healthcare Cost and Utilization Project National Inpatient Sample database (2009–2013) [9]. The estimated additional cost of dysphagia over the study period was $16.8 billion. Mean length of stay was 8.8 days for those with a dysphagia diagnosis versus 5.0 days for those without. Adult patients with dysphagia were 1.7 times more likely to die in hospital and 33% more likely to be discharged to a post-acute care facility than those without dysphagia. Similar results from a European study of dysphagia following acute ischemic stroke showed that patients with dysphagia more frequently had pneumonia (23.1% vs. 1.1%), higher mortality (13.6% vs. 1.6%), longer lengths of stay and were less frequently discharged to home (19.5% vs. 63.7%) [10].

Stroke practice guidelines recommend early screening for swallowing impairment [11–13]. For example, the 2018 American Heart Association/American Stroke Association guidelines for acute ischemic stroke recommend early screening to identify dysphagia and, for those in whom risk of dysphagia is identified, a swallowing assessment before the patient begins eating, drinking or receiving oral medications [11, 12]. Evidence suggests that formal screening programs are more effective at detecting dysphagia than informal approaches [14] and are effective for reducing pneumonia [15–17]. There is, however, a lack of agreement regarding optimal screening methodologies [18–28]. Most swallow screening protocols rely on observations by trained health care professionals who perform subjective evaluations of non-swallowing tasks (e.g., speech clarity, tongue motor function, voice quality and voluntary cough function) and swallows of water or other stimuli [23–28]. The detection of ≥ 1 sign(s) of concern has relatively good sensitivity (i.e., > 80%) for identifying patients in whom prior or subsequent videofluoroscopic or endoscopic examinations of swallowing confirm the presence of dysphagia or aspiration [24, 26–29]. Specificity is reported to range from 49 to 79%.

One acknowledged limitation of existing screening protocols is their reliance on observation of overt signs of aspiration; by definition, this results in poor sensitivity for identifying silent aspiration (i.e., aspiration without outward signs of difficulty), which is estimated to occur in up to 2/3 of stroke patients who aspirate [30, 31]. A further limitation is that the sensitivity and specificity of screening tests are typically determined through comparison of the net (i.e., worst) result observed across several screening criteria and the worst result obtained across several swallowing tasks in the reference assessment. Studies suggest that individuals who aspirate do not do so consistently across repeated presentations of similar boluses, even within a single examination [32, 33]. Such variability challenges the idea that the accuracy of screening test results can be properly evaluated through comparison to instrumental reference data collected on a separate occasion.

For the past few years, we have been developing a non-invasive medical device (the *Dysphagia Detection System*, DDS) to detect swallowing impairment based on automated machine analysis of cervical accelerometry signals, trained through direct comparison to simultaneous videofluoroscopy [34] (2012). If an accurate and reliable device can be developed, concerns regarding the reliance of swallow screening on subjective clinical observations would be obviated, together with the burden that currently exists for training health care professionals to recognize signs of swallowing impairment. We report the results of a prospective study to develop signal processing algorithms for the DDS device, with the ultimate goal of using this device to screen swallowing function in adults at risk of oropharyngeal dysphagia.

## Methods

This study involved prospective collection and classification of dual-axis (superior–inferior and anterior–posterior) accelerometry signals, collected from the front of the neck during swallows of water and of barium stimuli of different consistencies. These signals were collected with time-synchronized videofluoroscopy (VFSS), which was used as the clinical reference standard for determining true status (safe/unsafe; efficient/inefficient). The study was conducted at seven hospitals (six acute care; one inpatient rehabilitation hospital) between November 7, 2013, and March 11, 2015. The protocol was approved by the Institutional Review Board (IRB) of each participating medical center.

### Participants

Consenting participants were recruited from a broad population of adults considered at risk for non-congenital, non-surgical, and non-oncologic oropharyngeal dysphagia. The population included those with stroke or other brain injury aged ≥ 21 years and other inpatients or outpatients with dysphagia risk aged ≥ 50 years. Exclusion criteria included the presence of a nasogastric feeding tube; neck surgery (including tracheotomy within the past year, resection for oral or pharyngeal cancer, radical neck dissection, cervical spine surgery, carotid endarterectomy, orofacial reconstruction, pharyngoplasty, or thyroidectomy; routine tonsillectomy and/or adenoidectomy were not excluded); non-surgical trauma to the neck resulting in musculoskeletal or nerve injury; or radiation to the neck. These exclusions were applied due to the possibility that these conditions might alter or interfere with the ability to collect swallowing accelerometry signals using a sensor placed on the surface of the neck. Participants had to have sufficient cognitive function to be able to comply with study procedures.

### Investigational Device Description

The DDS is a portable, non-invasive device consisting of a dual-axis accelerometer (bandwidth up to 1600 Hz) contained in the plastic housing of a sensor unit that is attached by a single-use, disposable fixation unit to the front of a patient’s neck in midline, just below the palpable lower border of the thyroid cartilage. Vibrations are detected in the superior–inferior and anterior–posterior axes. The sensor unit was connected via a cable to an A/D converter (12-bit resolution, 10 kHz sampling frequency), which in turn was connected to a laptop computer where the signal was recorded.

### Procedures

The data collection protocol began by asking participants to swallow six comfortably sized sips of water from a cup. These water data were collected to demonstrate equivalence of the DDS signal profiles for water and thin-barium stimuli and will not be further discussed in this manuscript. The protocol continued with six sips of thin liquid barium, followed by three sips each of mildly thick, moderately thick and extremely thick barium. The thin and mildly thick liquids were taken by sip from 7-ounce cups containing 4 oz of liquid. The moderately thick and extremely thick barium were taken by teaspoon. Sip volume was measured based on pre- and post-sip cup weights. The thickened stimuli were prepared by mixing a powdered xanthan gum thickener (Nestlé Resource^®^ ThickenUp^®^ Clear™) with the thin liquid barium powder (Bracco Varibar^®^ Thin) and water in a 20% w/v barium concentration according to standard recipes (see Online Appendix for more details). Lateral view videofluoroscopy (recorded at 30 frames/second) and simultaneous accelerometry signals were captured on a laptop computer. Stopping rules were applied for safety with testing of a particular stimulus discontinued after two penetration–aspiration events and the entire protocol terminated after a total of five penetration–aspiration events.

### Videofluoroscopy Rating

All videofluoroscopy records were analyzed in a central lab utilizing a standard operating procedure (see Online Appendix for more details). Each bolus recording was randomly assigned to be assessed independently by two raters who were masked to the identity and diagnosis of the participant, study site, the bolus consistency and order of the bolus within the data collection sequence. The rating for each bolus included a measure of swallowing safety scored according to the 8-point Penetration–Aspiration Scale [1]; and pixel-based measures of residue severity taken on the last frame of each swallow [35]. When necessary, a meeting of at least three raters was convened to resolve discrepancies by consensus. If the raters concurred that visualization of the structures necessary for a particular rating was obscured, the feature in question was documented as not-rateable and became a missing data point, resulting in exclusion of that record from the data available for algorithm training. Confirmed ratings were transformed to binary scores as follows: for swallowing safety, 1–2 versus 3–8 on the Penetration–Aspiration Scale [1]; inefficiency was operationally defined as the presence of residue at the end of any swallow, filling ≥ 50% of the valleculae and/or the pyriform sinuses [36]. A participant was considered to have impaired swallowing safety (or efficiency) on a given consistency if at least one bolus in the series for that stimulus was rated as unsafe or inefficient, respectively.

### Accelerometry Signal Processing and Classifier Development

**Fig. 1:**
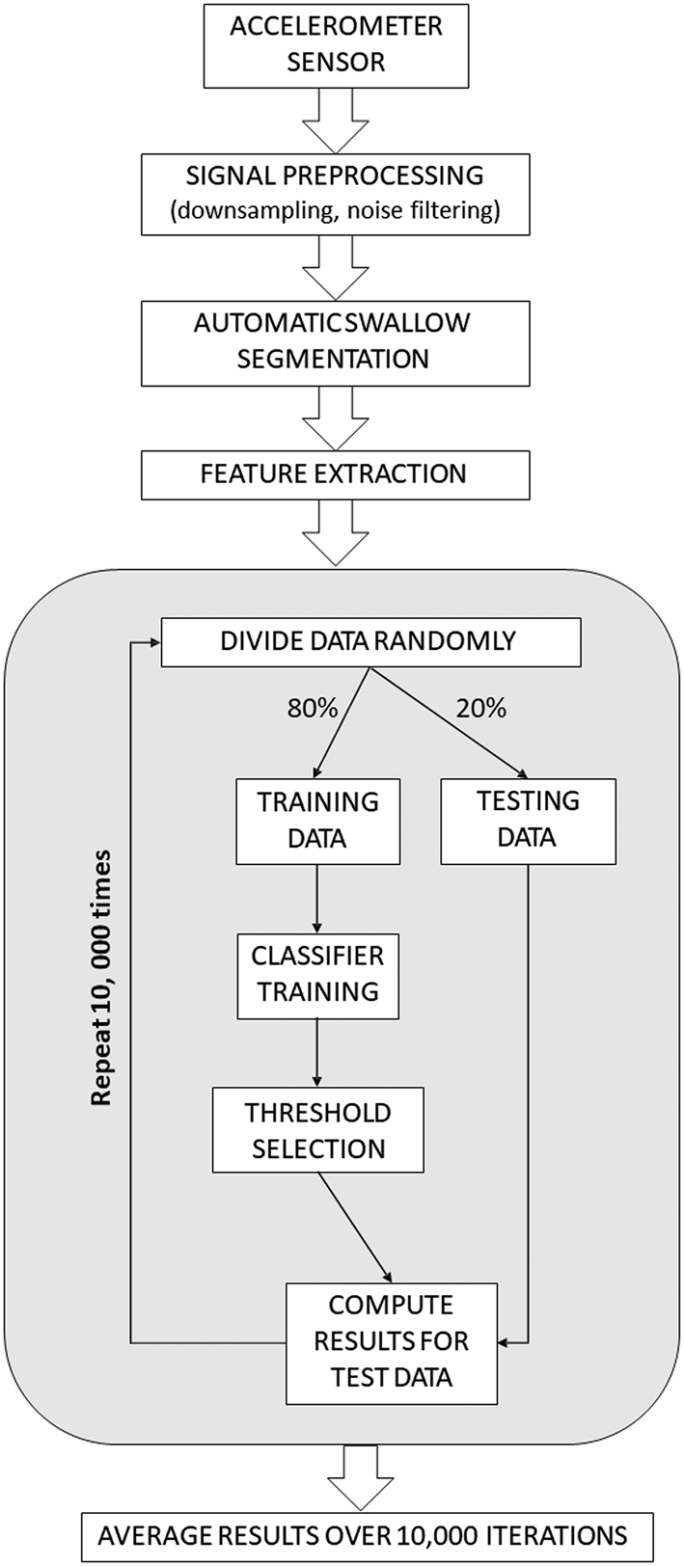
Flowchart showing the process used for developing classifier algorithms

The classifier development path is illustrated in Fig. 1. During preprocessing, the accelerometry signals were filtered first with a second-order high-pass Butterworth filter (0.1 Hz corner frequency) and then with a low-pass filter (1000 Hz corner frequency). This was followed by automated signal segmentation to isolate regions of swallow activity within each signal recording and feature extraction for analysis. Several well-established models for training classifiers were explored, including Support Vector Machine, Random Forest, Quadratic Discriminant Analysis and Linear Discriminant Analysis (LDA) [37]. Ultimately, a regularized LDA model approach using an equal covariance matrix for all estimated classes was selected because it had the most robust performance and a low risk of over-fitting to the training data [37]. Classifier models were built and validated in an iterative fashion using internal repeated random sub-sampling (also known as the Monte Carlo method [38, 39]). From the whole set of data, 20% of the participants were set aside as a validation set and the signals from the remaining 80% of participants were used to train the classifier (training set). The trained classifier was then used to estimate the classes (i.e., safe, unsafe; efficient, inefficient) of the cases in the validation set that were previously unseen by the classifier. These results were then compared to the true classes obtained from the videofluoroscopy analysis. This process was repeated 10,000 times with randomly selected validation and training sets and the classifier was re-generated during each iteration with the new training set. In this manner, each independent iteration could be considered analogous to training the device on a sample of approximately 244 individuals, representing 80% of individuals in a particular cohort in a particular setting, before being applied unsupervised to the remaining 20% of unseen patients (approximately 61) in that cohort and setting. Random splitting of the data was stratified by the participant’s status derived from the VFSS results; thus, all boluses from a given participant were either in the training or the validation set on a given iteration. The area under the curve (AUC) measures of the receiver operator curve (ROC) from each of the independent validation sets across all runs were averaged to provide the mean and standard deviation of the AUC for the classifier at the bolus level. The large number of iterations (i.e., 10,000) was chosen in light of the high standard deviation of the resulting AUC.

In total, six different LDA classifiers were developed: three algorithms to detect impaired swallowing safety (thin liquids; mildly thick liquids; moderately thick liquids) and three to detect impaired swallowing efficiency (thin, mildly and moderately thick liquids). Given the limited availability of impaired swallowing safety data for the moderately thick consistency, it was decided to use combined moderately and extremely thick stimuli in the training set of the moderately thick safety classifier; the corresponding validation sets included only moderately thick consistency swallows.

Analysis of the cumulative frequency of impaired swallowing safety by bolus number, across the series of thin liquid boluses administered, showed new occurrences beyond the first bolus (7.5%) to the second (15%), third (18.5%) and fourth bolus (22.2%), with relatively small incremental detection rates on the fifth (23.4%) and sixth (25.6%) boluses. Given this finding, the mean predicted probability of impairment was summarized at the participant level using up to four boluses for thin and up to three boluses for other consistencies. The ROC at participant level was obtained by comparing these mean predicted probabilities with the participant level class label obtained using the “at least one positive” roll-up rule on the VFSS binary data. Thus, if the VFSS showed a problem on at least one bolus of a given consistency, that participant was classified as having impaired swallowing function on that consistency.

## Results

### Participants

**Fig. 2:**
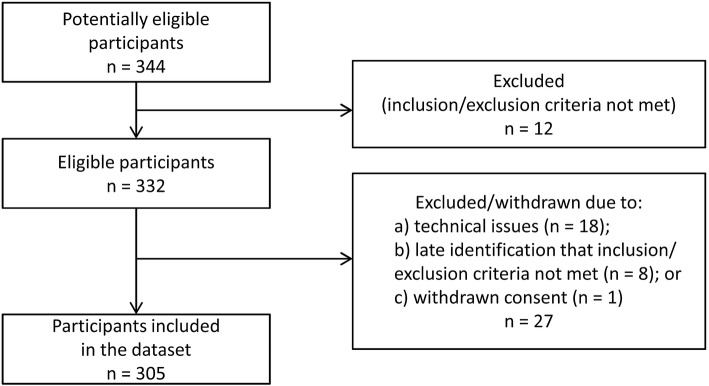
Overview of participants in the study

A total of 344 patients consented to participate in the study. Of these, 12 were excluded based on protocol requirements (see Fig. 2). Videofluoroscopy was performed in 332 participants for whom the demographic characteristics are summarized in Table 1. Complete VFSS and DDS signal data were available for at least 2 boluses for 305 participants. There were no serious adverse events related to the device or the swallowing trial protocol.

**Table 1:** Demographic characteristics of the subjects who underwent videofluoroscopy by diagnostic subgroup

	N	Stroke	Other brain injury	Other, aged ≥ 50	Combined
		(*N* = 107)	(*N* = 18)	(*N* = 207)	(*N* = 332)
Sex	332				
Women		48 (45%)	4 (22%)	113 (55%)	165 (50%)
Men		59 (55%)	14 (78%)	94 (45%)	167 (50%)
Age	332	70 ± 14	67 ± 11	73 ± 11	72 ± 12
VFSS recorded	332				

The data are shown according to the following convention *x* ± *s* represents the mean ± one standard deviation

### Prevalence of Impaired Swallowing Safety and Efficiency in the Study Population

Videofluoroscopy data for 1730 thin, 872 mildly thick, 833 moderately thick and 794 extremely thick boluses were analyzed. Table 2 shows the frequencies of unsafe swallows and efficiency issues by consistency at both the bolus and the participant levels. The number of available data points differs for the safety versus efficiency ratings because the visibility of the airway (necessary for rating safety) on a given recording may have differed from visibility of the valleculae and pyriform sinuses required for rating efficiency. Post hoc examination of the frequency distribution of Penetration–Aspiration Scale rating data by the central laboratory showed that 23 participants (i.e., 7.5%) had silent aspiration (i.e., PAS scores of 8) on at least one thin liquid bolus. The participant-level prevalence of silent aspiration dropped to 5.3% on the mildly thick liquids and < 1% on the moderately and extremely thick liquids.

**Table 2:** Prevalence of impaired swallowing safety and impaired swallowing efficiency at the bolus and subject level, by stimulus type

Stimulus consistency	Number of data points available		Impaired safety				Impaired efficiency			
	Boluses	Participants	Bolus level		Participant level		Bolus level		Participant level	
			*n*	%	*n*	%	*n*	%	*n*	%
Thin (1–6 boluses)	1730	305	125	7.2	70	23.0	115	6.7	60	19.7
Mildly thick	872	302	51	5.9	42	13.9	86	9.9	54	17.9
Moderately thick	833	281	17	2.0	14	5.0	75	8.9	51	18.2
Extremely thick	794	268	11	1.4	10	3.7	67	8.4	48	17.9

### Accuracy of Classifiers

Tables 3 and 4 summarize the estimated accuracy of the DDS classifiers for swallowing safety and efficiency, respectively. For the primary outcome of detecting impaired swallowing safety on thin liquids, a mean AUC of 80.9% on the ROC was obtained at the bolus level. At the participant level, the mean AUC was 81.5%, with sensitivity (i.e., true positive rate) of 90.4% and specificity (i.e., true negative rate) of 60.0%. Classifier performance was similar for detecting impaired swallowing safety on thicker consistencies. The efficiency classifiers achieved sensitivities of ~ 80% and specificities of 60% across the consistencies tested.

**Table 3:** Classifier accuracy for detecting swallow safety problems by consistency

Consistency	Bolus level AUC (%) mean ± SD	Participant level AUC (%) mean ± SD	Sensitivity (%) mean ± SD	Specificity (%) mean ± SD
Thin	80.9 ± 5.9	81.5 ± 6.1	90.4 ± 7.7	60.0 ± 7.8
Mildly thick	83.9 ± 5.6	83.6 ± 5.9	92.7 ± 8.7	59.9 ± 7.5
Moderately thick^a^	78.9 ± 11.9	79.7 ± 15.1	89.1 ± 22.0	59.6 ± 7.6

^a^Data for the extremely thick consistency were included in the training set for the moderately thick safety model

**Table 4:** Classifier accuracy for detecting swallow efficiency problems by consistency

Consistency	Bolus level AUC (%) mean ± SD	Participant level AUC (%) mean ± SD	Sensitivity (%) mean ± SD	Specificity (%) mean ± SD
Thin	76.7 ± 6.4	77.7 ± 7.5	82.3 ± 11.0	59.2 ± 7.9
Mildly thick	80.1 ± 6.0	78.0 ± 7.7	82.4 ± 11.0	59.6 ± 7.9
Moderately thick	73.3 ± 7.1	71.9 ± 8.3	79.3 ± 11.7	59.3 ± 8.2

## Discussion

In this study, signal processing classifiers were trained using a large dataset collected from a heterogeneous sample of individuals with risk for dysphagia. As the first step in identifying dysphagia, it is desirable for swallow screening methods to have wide applicability and accuracy in patient populations with dysphagia risk related to varied medical conditions and for whom the pathophysiological mechanisms leading to impaired swallowing safety or efficiency may be heterogeneous. Within this broader objective, the inclusion criteria for this particular study excluded individuals with oncological, structural or congenital etiologies of dysphagia, and the definition of impaired swallowing safety was set conservatively as a Penetration–Aspiration Scale score ≥ 3.

The DDS classifiers developed in this study were able to identify impaired swallowing safety on thin liquid swallows with high accuracy. In clinical practice, the achieved classifier performance results would translate to failure to identify impaired swallowing safety in 10% of patients and over-detection in 40%. This degree of over-detection is acceptable in screening, provided that referral for assessment to verify the patient’s swallowing status occurs in a timely manner [40, 41].

Several differences between the classifier results of this study and commonly used swallow screening protocols should be noted. First, the results for thin liquids in this study were obtained using a small number of sips (i.e., 4). Second, the results obtained in this project are consistency specific. It is encouraging that similar algorithm performance was achieved for detecting impaired swallowing safety across a range of consistencies. The current data are consistent with previous studies in showing reduced rates of penetration–aspiration with thickened liquids [42–45]. However, this also meant that a smaller number of impaired signal examples were available for algorithm training for moderately thick liquids. Additionally, protocol-mandated stopping rules did not permit more severely affected participants to receive the thicker consistencies.

Our data corroborate observations from previous studies that the occurrence of penetration–aspiration on a given consistency varies from bolus to bolus in individuals with impaired swallowing safety [32, 33]. This finding has two major implications: (1) more than one swallow should be assessed to determine if a patient can swallow a given consistency safely; and (2) validation of the accuracy of any method for detecting swallowing impairment at the bedside requires direct comparison to a simultaneous reference standard rather than a reference test performed at a different time [26–28]. An advantage of using direct comparison to simultaneous videofluoroscopy is that we were able to include verified examples of silent aspiration in the training sets of unsafe swallows in this study.

Given the overall objective of developing a swallow screening instrument that would have broad utility to identify impaired swallowing safety in a heterogeneous sample of patients, no attempts were made to stratify the data by PAS severity beyond the binary groupings of < versus ≥ 3. Similarly, we did not limit the examples of impaired swallowing safety to cases of airway invasion before, during or after the swallow. Although previous studies have identified links between specific swallowing parameters (such as measures of hyolaryngeal excursion) and the features of swallowing accelerometry signals [46, 47], the algorithm training process in this study allowed for a variety of pathophysiological mechanisms to be included in the subset of cases that the device learned to identify as impaired. The study is not powered to allow stratification by severity, timing or mechanism of swallowing impairment; stratifications of this sort would require much larger datasets with adequate representation of the different groupings of interest.

The majority of swallow screening protocols in current use focus on signs of impaired safety and risk of aspiration, without consideration of swallow efficiency. Given this, it is encouraging that sensitivities and specificities of ~ 80 and 60%, respectively, were also obtained for the separate classifier algorithms developed to detect impaired swallowing efficiency using the DDS. Individuals who have impaired swallowing efficiency may require more time to complete a meal, and are thought to be at risk both for post-swallow aspiration and malnutrition [6, 36]. Our data show that swallowing inefficiency is a common finding. Furthermore, the data suggest that residue can be common after swallows of thin liquids. We found no evidence to support the widespread assumption that residue increases with thicker stimuli (Table 2). This finding may be explained by the use of a xanthan gum thickener rather than starch-based thickeners [48], which are commonly used in clinical practice.

## Conclusion

In this study, we generated accelerometry signal classification algorithms to detect impaired swallowing safety in patients at risk for dysphagia with high accuracy using a pragmatic DDS protocol involving a series of four thin liquid sips. Additional algorithms to detect impaired swallowing safety with thicker consistencies and to detect impaired swallowing efficiency across the range from thin to moderately thick liquids were also developed. Development of these algorithms represents a critical first step in the process of developing an accurate, non-invasive, accelerometry-based device for swallow screening. The next step will be to validate these algorithms in a prospective study using a novel patient sample. Based on the current study results, a validation study of this sort will require simultaneous collection of screening and reference data. If such a validation study were to be performed with a fixed design in a population with 60% participant-level prevalence of impaired swallowing safety on thin liquids, with at least 30% of the thin liquid swallows displaying the swallowing safety problem in those participants, power calculations based on the results of this study suggest that a minimum sample of 500–600 participants would be required, assuming performance targets of 86% sensitivity, 60% specificity and 90% power. If accurate device performance can be confirmed for detecting impaired swallowing safety and/or impaired swallowing efficiency on a range of liquid consistencies in the broad population of individuals at risk for dysphagia, comparisons to current screening methods, which rely on subjective clinical observations of swallowing difficulty, will also be warranted.

## Electronic supplementary material

Below is the link to the electronic supplementary material.

Supplementary material 1 (DOCX 46 kb)
