# Supplementary material for: Development of a Non-invasive Device for Swallow Screening in Patients at Risk of Oropharyngeal Dysphagia: Results from a Prospective Exploratory Study
Source: Dysphagia. 2019 Jan 5;34(5):698–707. doi: 10.1007/s00455-018-09974-5 (PMC6717605; doi:10.1007/s00455-018-09974-5)
Supplement: Supplementary file 1 — Supplementary material 1 (DOCX 46 kb) [file 455_2018_9974_MOESM1_ESM.docx]

**APPENDIX**

Development of a Non-invasive Device for Swallow Screening in Patients at Risk of Oropharyngeal Dysphagia: Results from a Prospective Exploratory StudyStimuli

1. 1. Stimuli

Liquid barium was prepared in four consistencies for the study (thin, mildly thick, moderately thick and extremely thick) using bottled spring water (Nestlé Ice Mountain®), barium sulfate powder (Bracco Varibar® Thin Liquid) and, for the thickened stimuli, a xanthan gum thickener (Nestlé ThickenUp Clear®). A barium concentration of 20% w/v was used for all consistencies. A standard operating procedure for mixing the stimuli was followed. Ingredients were weighed on an OHAUS PA1502 Pioneer Precision Balance. Wet and dry ingredients were combined using a Bosch MUM4405 Compact Tilt-Head Stand Mixer. Recipes for these stimuli were as follows:

| Thickness | Expected Solution Volume (mL) | Mass of Water (g) | ThickenUp Clear® powder (g) | Varibar® Thin Liquid powder (g) |
| --- | --- | --- | --- | --- |
| Thin | 400 | 375 | none | 105.6 |
| Thin | 800 | 750 | none | 211 |
| Mildly-Thick | 400 | 375 | 3.6 | 105.6 |
| Mildly-Thick | 800 | 750 | 7.2 | 211 |
| Moderately-Thick | 200 | 188 | 4.0 | 52.8 |
| Extremely-Thick | 200 | 188 | 5.8 | 52.8 |

1. Videofluoroscopy Rating

A standard operating procedure (SOP) was used for rating the videofluoroscopy recordings for this study, in order to yield reliable measures of swallowing safety using the 8-point Penetration-Aspiration Scale [1] and both ordinal and pixel-based measures of swallowing efficiency [33, 34].

*VFSS Recording Review and Clipping*

Upon receipt of a videofluoroscopy recording at the core lab, an initial review was performed by one of the lab engineers to confirm adequate image quality and temporal resolution (frames per second) for analysis. Each full-length recording was reviewed to identify the time codes associated with onset/offset of the fluoro x-ray for each sip or bolus contained in the recording. The boundaries identified were then used to splice the original full-length recording into smaller video clips, each containing the swallows associated with a single bolus. These boundaries were entered into a spreadsheet which was then passed to MatLab for video clipping. The spliced bolus-level video clips, with no audio track, were labelled with a random file number, with the master key retained in a file on the lab research server. Bolus-level video clips were generated in sets of 150 which were referred to as “batches” of videos.

*Video Rating Assignment*

Each bolus level video clip was randomly assigned to two raters. Raters were licensed Speech Language Pathologists (SLPs) who had completed a training program, and had demonstrated competency in all required rating procedures.

*VFSS Analysis and Discrepancy Resolution*

The raters made measurements of swallowing safety and efficiency for every swallow contained in each bolus level video clip. Raters were provided a computer with Windows 7 operating system and two monitors (one for video viewing and another for data entry). The 64-bit ImageJ software (National Institutes of Health, https://imagej.nih.gov) was used to review each bolus level video clip. ImageJ allows the users to view the videos in real-time as well as frame-by-frame (forward frame advancement as well as backwards, if this is felt to be helpful). Raters were given the freedom to review the video clips as many times as they wished. Raters were instructed to avoid ImageJ contrast adjustment and enhancement tools.

When a bolus clip was first opened for review, the rater was asked to review the entire bolus level video clip using frame-by-frame viewing software to identify the number of swallow(s). Swallowing safety and efficiency measures were then completed for each subswallow in the video clip. Raters were instructed that any field or variable could be marked with a dot (i.e., “.”) at their discretion, if they deemed the measure “unrateable” (e.g., obstructed visibility, quality too poor, late fluoroscopy, missed event, etc.).

Swallowing safety was scored using the 8-point Penetration-Aspiration Scale [1] (PAS):

1 = Material does not enter airway

2 = Material enters the airway, remains above the vocal folds, and is ejected from the airway

3 = Material enters the airway, remains above the vocal folds, and is not ejected from the airway

4 = Material enters the airway, contacts the vocal folds, and is ejected from the airway

5 = Material enters the airway, contacts the vocal folds, and is not ejected from the airway

6 = Material enters the airway, passes below the vocal folds and is ejected into the larynx or out of the airway

7 = Material enters the airway, passes below the vocal folds, and is not ejected from the trachea despite effort

8 = Material enters the airway, passes below the vocal folds, and no effort is made to eject

Swallowing efficiency was determined based on three criteria:

a) the number of subswallows that occur for a single bolus;

b) ordinal measures of residue that remains in the valleculae and pyriform sinuses at the end of the swallow; and

c) pixel-based measures of residue that remains behind in the valleculae and pyriform sinuses at the end of the swallow.

Residue measurements were made on the terminal video frame for each subswallow in the video clip. To identify this terminal event frame, raters used the following definition: the first frame at which the pyriform sinuses are seen in their lowest position (relative to the spine) that occurs before *all* of the following:

• End of video clip

• End of fluoro

• Onset of a subsequent swallow as indicated by rapid superior-anterior movement of the hyoid bone

• Non-swallow events (e.g., UES re-opening, coughing, tongue pumping to prepare the subsequent bolus)

If the view of the pyriform sinus was obstructed (e.g., behind a raised shoulder shadow), raters were instructed to select the frame immediately before the view became obstructed. If this earlier frame preceded visible relaxation of the pharynx, residue was considered unrateable.

Ordinal ratings of residue severity were recorded separately for the vallecular space and pyriform sinus for each subswallow. Residue ratings were initially made using a 4-point ordinal scale based on the scale developed by Eisenhuber and colleagues [34].

0 = no residue (coating of structures is considered no residue, must ‘pool’)

1 = ‘mild’ residue (the level of contrast material constitutes < 25% of the height of the structure)

2 = ‘moderate’ residue (the level of contrast material is > 25% and < 50% of the height of the structure)

3 = ‘severe’ residue (the level of barium is > than 50% of the height of the structure)

In order to generate more precise measures of residue severity, measures of the degree to which the valleculae and pyriform sinuses were full (%-full) were performed using pixel-tracing in ImageJ software. For these measures, the area of the residue was traced and compared to the area of the space (valleculae or pyriform sinuses).

*Agreement and Discrepancy Resolution*

- For Penetration-Aspiration Scale ratings, absolute agreement across blinded duplicate ratings was required. Whenever a difference in the PAS rating occurred, the video clip was flagged for review in a discrepancy resolution meeting.
- For frame or event selection, acceptable agreement was considered to have been achieved when events did not differ by more than 5 frames (i.e., 167 ms) across raters. With respect to identifying the terminal frame of each swallow to be used for residue measurement, provided this criterion had been met, the latest frame across raters was chosen as the frame of record by convention. When differences of greater than 5 frames occurred across raters for event identification, the video clip was flagged for review in a discrepancy resolution meeting.
- For ordinal ratings of residue, absolute agreement across duplicate ratings was required. Whenever a difference in an ordinal scale rating occurred, the video clip is flagged for review in a discrepancy resolution meeting.
- For pixel-based measures of residue, the difference pixel area was divided by the average of the two scores. If the resulting ratio for each space (valleculae; pyriform sinus) was less than 1.6, the lowest value (i.e., the minimum estimate of impairment) was recorded as the rating of record. If the resulting ratio for each space (valleculae; pyriform sinus) was greater than 1.6, the video clip was flagged for review in a discrepancy resolution meeting.

At each discrepancy resolution meeting, a minimum of 3 trained raters were present. These raters may or may not have included the same individuals who made the first, discrepant ratings. In these meetings, the clip was displayed on a large monitor, reviewed, and each of the raters present independently documented a new rating of the discrepant parameter (frame selection, penetration-aspiration scale, ordinal residue ratings or pixel-based residue ratings). These new ratings were then openly declared. If the new ratings were not in unanimous agreement, the case was further reviewed and discussed until all raters present achieved consensus on the score that should apply.

*Transformation of Scores to Binary Class Labels*

Penetration-aspiration scale scores of 1 and 2 were considered to be safe. Scores of 3 and higher were considered unsafe. For this particular project, the worst safety score across all subswallows was reported for each bolus. In the event that a safety score could not be provided for one or more subswallows and all other subswallows in the video clips were considered normal, the overall bolus results are marked as missing.

Pixel-based residue ratings of 50% full or worse were considered inefficient. In the event that pixel tracing was not possible due to an obstructed view, the ordinal residue rating scales were considered. Ordinal residue ratings of 3 or higher were considered inefficient, reflecting residue occupying > 50% of the available space in either the valleculae or the pyriform sinuses. For this particular project, the worst efficiency score across all subswallows was reported for each bolus. In the event that an efficiency score could not be provided for one or more subswallows and all other subswallows in the video clips were considered normal, the overall bolus results were marked as missing.
